# Tube Exposure Repair

**DOI:** 10.5005/jp-journals-10008-1121

**Published:** 2012-10-16

**Authors:** Stirbu Oana, Jorge Vila

**Affiliations:** Consultant, Barcelona Institute of Ophthalmology, Barcelona, Spain; Consultant, Clinical University Hospital, Valencia, Spain

**Keywords:** Conjunctival erosion, Patch graft, Tube shunt.

## Abstract

All across the world, glaucomatologists are adopting broader use of glaucoma drainage implants even as a primary surgical modality. To avoid tube exposure, which may predispose the eye to endophthalmitis, the implanted tube must be covered by a patch graft. However, these patch grafts also carry a high rate of progressive thinning and erosion, which is believed to result from the lack of cellular infiltration from the surrounding host conjunctival stroma and poor integration of these patch grafts to the host tissue. An ideal patch graft should offer good tensile strength, be suitable for tectonic support, and have biological activities to promote cellular infiltration by the surrounding host conjunctival stroma, thus reducing progressive allogeneic patch graft thinning/erosion. This review talks about various materials and modalities used for an exposed tube repair.

**How to cite this article:** Oana S, Vila J. Tube Exposure Repair. J Current Glau Prac 2012;6(3):139-142.

## INTRODUCTION

More than three decades after the addition of tubes in the glaucoma surgical armamentarium, tube implantation technique (known under several names, such as tube shunt, aqueous shunt, aqueous tube shunt, seton, glaucoma drainage device, glaucoma drainage implant, glaucoma drainage shunt, etc.) has gained numerous adepts worldwide and various designs have been invented in order to improve its efficacy and lower the complications.

The growing confidence in glaucoma drainage device implants is accredited by the recent results of the tube *vs* trabeculectomy study. After 5 years of follow-up, shunt surgery proved a higher success rate compared to trabeculectomy with mitomycin C (MMC). Both procedures were associated with similar reduction of the intraocular pressure (IOP) and use of supplemental medical therapy, while additional glaucoma surgery was needed more frequently after trabeculectomy with MMC than tube shunt placement.^1^

The complications described after tube surgery include: Immediate hypotony after surgery, excessive capsule fibrosis with decreased permeability of the capsule formed around the plate, erosion of the tube or plate edge, strabismus and infection. The most common shunt-specific delayed complication is exposure of the tube through overlying eroded conjunctiva.^2^

### Tube Exposure

The causes of the conjunctival erosion are not well-defined. Early tube exteriorization is usually related to a dehiscence of the suture, while late onset tube extrusion is produced by erosion of the scleral/graft patch and of the overlying conjunctiva in the context of a quiet eye. One mechanical factor that might influence is the micromotion of the tube with ocular movements and blinking, in a pathologic ocular tissue or in elder patients, in which thinning and micro-filtration lead to hypotonia with its entire syndromic picture.

The tube of a glaucoma drainage implant must not be left in direct contact with the overlying conjunctiva. The use of patches or grafts of different materials has improved the development of this technique to reduce the frequency of endophthalmitis.^3^ Various authors have proposed different tissues for the graft with successful results: Autologous or human donor sclera,^4^ pericardium,^5^ dura mater,^6^ cornea or human cadaveric fascia lata,^7^ amniotic membrane,^8^ autologous Tenon^9^ and expanded polytetra-fluoroethylene.^10^

In the late 90s, various case reports have drawn the attention on the possibility of patch melting, involving different tissues, such as pericardium patch graft^11^ or dura mater.^6^ A comparative study of the tube coverage on 62 eyes with donor sclera, dura mater or pericardium patch grafts in the initial surgery showed that no material was more prone to melting than the other.^12^

The frequency of tube extrusion when Ahmed device is implanted varies from 5 to 7% to 14.3%^13-15^ and in a recently published study on 12 patients, the authors report a 30.8% of tube exposure.^16^ Nevertheless, these high frequencies are not present in multicenter or meta-analysis studies.

In a multicenter study on 276 patients which determined the relative efficacy and complications of the Ahmed FP7 glaucoma valve and the Baerveldt 101 to 350 glaucoma implant in refractory glaucoma, the authors present a frequency of tube erosion of 2% in Ahmed device and 3% in Baerveldt implant at 1 year follow-up. Interestingly, no tube erosion was depicted as early postoperative complication, defined as occurring in the first 3 months after tube implantation.^17^

A meta-analysis of 38 studies including 3,255 eyes with an average follow-up of 26.1 ± 3.3 months evaluated conjunctival erosion and tube exposure in Ahmed, Baerveldt and Molteno implants. The overall incidence of exposure was 2.0 ± 2.6% (n = 64) of eyes with an average exposure/month of 0.09 ± 0.14%. Among individual drainage devices, the authors did not find significant differences in the incidence of exposure or percent exposure per month.^18^

As for the early onset of the leak, between postoperative months 1 and 3, few studies approached the topic. One of them includes three patients with iridocorneal endothelial syndrome and one patient with chronic panuveitis, on which uneventful implantation of Molteno (1), Ahmed (2), and Baerveldt (1) aqueous shunts was performed. The use of aqueous suppressants, bandage contact lens, conjunctival photocoagulation, sutures, tissue adhesives, conjunctival autografts and scleral patch grafts were unsuccessful in the described cases. The authors described resolution of the leak after removal of the aqueous shunt.^19^

Notably, good results were reported with a modified implant technique in which the need for tube coverage patch is obviated. In a retrospective study on 35 consecutive patients who underwent Molteno implant with a partial thickness scleral tunnel to cover the tube, conjunctival dehiscence and tube exposure was not observed in the 4-year follow-up period. The authors suggest that outcome may be related to an absence of the immune-mediated processes proposed for thinning and melting of patch grafts.^20^

### Tube Exposure Repair

The externalization or extrusion of the tube through an eroded conjunctiva represents one of the most serious complications of the glaucoma drainage device, as it seems to represent a major risk factor for endophthalmitis.^21^ When tube exposure occurs, prompt surgical revision is highly recommended to prevent this potentially devastating complication. Endophthalmitis after aqueous drainage device may occur in the first 2 to 30 days postoperatively^22,23^ or in the late postoperative period, related to the tube externalization^24-26^and its frequency varies from 0.9 to 6.3%.^27,28^ Interestingly, a report of a case of late onset *H. influenzae* endophthalmitis in an immunized child after bilateral glaucoma drainage implants without evidence of conjunctival erosion or wound dehiscence was found in the literature.^29^ In a study on late endophthalmitis associated with Baerveldt glaucoma implants, exposure of the glaucoma drainage implant tube was present in all cases and the authors recommended prophylactic surgical revision with a patch graft in all cases in which there is an exposed tube.^30^

No author cited below advocates simple conjunctival closure without a graft; the graft materials used to repair the tube extrusion are mainly the same used to initially cover the drainage implant tube (Table 1).

**Table 1:** **Table 1:** Materials used for tube coverage and/or tube exposure repair

		**Ocular tissues**	
Autologous		Conjuntiva, tenon, cornea, sclera, collagen fibrous tissue	
Heterologous		Sclera, cornea	
		**Extraocular tissues**	
Autologous		Fascia lata, buccal mucosa	
Heterologous		Dura mater, pericardium, acellular dermis, amniotic membrane	
		**Synthetic tissues** Ologen^®^, polytetrafluoroethylene	

The graft tissues reported to have been used in the tube exposure repair include autologous and donor eye tissues (full thickness sclera,^11^ split-thickness hinged scleral flap^31^ cornea,^32^ autologous scar tissue,^33^ conjunctival pedicle flaps^34^), extraocular tissues (amniotic membrane,^35^ donor acellular dermis,^36^ buccal mucosa^37^) and synthetic materials such as biodegradable scaffold collagen matrix Ologen^®^.^38^

In a case series of two patients presenting tube erosion after Ahmed and Baerveldt implant respectively, with original pericardial patch graft, a full thickness donor scleral graft was used to cover the tube.^11^ A conjunctival pedicle flap attached to its original vascular supply was used to cover tube exposure in 4 eyes with Baervaldt implant, in addition to a new human pericardium graft.^34^ Erosion of the drainage tube was reported to be successfully managed using a double layer of amniotic membrane in a consecutive series of three patients with exposure of the tube secondary to necrosis of the overlying bovine pericardial patch and conjunctiva. The repair of the defect was carried out with a double layer of amniotic membrane, the inner one acting as a graft and the outer as a patch. The authors also used autologous serum postoperatively to promote epithelial growth.^35^ A retrospective review of 8 eyes that underwent corneal patch graft repair of exposed tubes showed stable conjunctival coverage with no epithelial breakdown over the corneal patch graft and with no tube reexposure, scleral thinning or ocular infection in 7 eyes.^32^ The use of oral buccal mucous membrane in combination with a lamellar corneal patch graft for repair of 3 exposed tubes was described in cases with limited supply of local conjunctiva.^37^ A recent and highly recommended study details a new surgical technique of repairing tube erosion using donor acellular dermis graft, as also the complications of the repair surgery. The author used the dermis graft in 30 cases, leaving a 2 to 3 mm intentional gap between the conjunctival edges over the dermis graft, with complete epithelization within weeks.^36^ Also, a split-thickness hinged scleral flap technique resulted in the successful recovery of the tube without reexposure or serious complications in 3 cases.^31^A recent case report presented Ologen^®^, a biodegradable collagen matrix implant that induces normalized wound healing, successfully used in one patient with Baerveldt tube exposure.^38^

## CONCLUSION

Conjunctiva erosion and tube exposure are infrequent complications of glaucoma drainage implant surgery, which need prompt repair due to the risk of endophthalmitis.

While many techniques and materials are successfully used to address this situation, further comparative prospective studies are required to determine the best repair method.
